# The Epidemiology and Clinical Presentation of the Acute Imbalance Syndrome (AIS)—A Systematic Review and Meta‐Analysis

**DOI:** 10.1111/ene.70651

**Published:** 2026-06-01

**Authors:** Konstantin Schmidt, Andreas Zwergal, Diego Kaski, Alexander Andrea Tarnutzer

**Affiliations:** ^1^ Faculty of Medicine University of Zurich Zurich Switzerland; ^2^ Department of Neurology LMU University Hospital, LMU Munich Munich Germany; ^3^ German Center for Vertigo and Balance Disorders (DSGZ) LMU University Hospital, LMU Munich Munich Germany; ^4^ SENSE Research Unit, Department of Clinical and Movement Neurosciences UCL London UK; ^5^ Neurology Cantonal Hospital of Baden Baden Switzerland

**Keywords:** acute imbalance syndrome, dizziness, epidemiology, gait ataxia, nystagmus, vertigo

## Abstract

**Objectives:**

The acute imbalance syndrome (AIS) refers to acute‐onset and persistent vertigo, dizziness and/or imbalance without nystagmus, reflecting a subset of the acute vestibular syndrome (AVS) with or without nystagmus. While AVS with nystagmus is well characterized and the approach to these patients is validated, much less is known about patients presenting with AIS. This systematic review synthesizes current epidemiologic and clinical evidence on AIS.

**Methods:**

MEDLINE and Embase (2000–2024) were searched for English‐language original articles addressing acute imbalance, vertigo/dizziness and reporting on nystagmus and/or AVS/AIS. Two reviewers independently screened articles and extracted predefined parameters. The review was registered with PROSPERO (CRD42024623068).

**Results:**

Forty studies (*n* = 11,731 patients) were included, with 2730 patients being classified as AVS. Prevalence of AVS (with/without nystagmus) amongst all acutely dizzy patients was 16.4% and 50.5%, respectively, in two studies. 49.4% of central AVS cases were classified as AIS. Whereas AIS presentation was rare in AICA strokes (9%), fractions reached 40% (PICA), 57% (SCA), and 62% (PCA) for other vascular territories. Data on underlying causes in noncentral AIS was limited, with dysautonomia, drug intoxication, and heart disease being most frequently identified.

**Conclusions:**

With almost 50% of central AVS cases presenting as AIS, this emphasizes the importance of selecting bedside testing appropriately, focusing on algorithms such as STANDING or graded truncal instability. Patients presenting with AIS have a distinct differential diagnosis than those with AVS with nystagmus, mostly due to the distribution of noncentral causes. Promoting awareness of AIS and its diagnostic approach should be prioritized in emergency and acute neurological settings.

## 1 Introduction

Acute dizziness, vertigo, and imbalance are frequent reasons for presentation to the emergency department (ED), accounting for approximately 2%–5% of all ED visits [1, 2, 3]. Although many cases are ultimately benign, cerebrovascular causes must always be considered, even when symptoms occur in isolation [4]. Given the large number of affected patients, the management of these cases represents a major diagnostic and economic challenge for acute healthcare systems [3, 4].

Combined bedside ocular motor testing as implemented by the HINTS (Head Impulse, Nystagmus, Test of Skew) [5] is highly accurate in patients presenting with acute‐onset persistent vertigo or dizziness, gait imbalance, nausea/vomiting, motion intolerance and nystagmus, with a sensitivity of 95.3% (95% confidence interval [CI] = 92.5%–98.1%) and a specificity of 92.6% (88.6%–96.5%) [6]. In 1988, Hotson and Baloh firstly termed this combination of findings (including the presence of nystagmus) as acute vestibular syndrome (AVS) and related it to an acute vestibular tone imbalance caused by a rapid, unilateral lesion to either peripheral or central vestibular structures [7]. Noteworthy, in the definition of AVS as proposed in the 11th edition of the International Classification of Diseases (ICD‐11), the presence of nystagmus became nonmandatory [8], thus broadening the spectrum of presentations meeting diagnostic criteria of an AVS, but also expanding its pathophysiological origin beyond acute unilateral vestibular lesions. Consequently, the diagnostic approach to AVS needs refinement for cases without nystagmus, as algorithms are optimized to the “classical AVS” (with nystagmus).

To date, the HINTS have been validated only in a single‐center cohort of acutely dizzy patients demonstrating no nystagmus and showed a quite poor diagnostic accuracy of 66.7% for the separation of peripheral and central cases [9]. This finding is expected, as HINTS criteria majorly rely on signs of asymmetric vestibular signal input, such as vestibular‐ocular reflex asymmetry or skew deviation as part of an ocular tilt reaction. Nonvestibular causes thus will be classified as central due to a bilaterally normal HIT in the absence of skew deviation or gaze‐evoked nystagmus. Notably, the absence of nystagmus is frequently documented in patients with nonvestibular causes of vertigo or dizziness, highlighting a potential source of misdiagnosis [1, 10, 11]. At the same time, however, a significant fraction of patients with central (ischemic) causes of acute prolonged vertigo or dizziness may demonstrate no spontaneous nystagmus (56.4% [12]) or any nystagmus at all (including gaze‐evoked nystagmus) as reported by Nikles and colleagues in 50% of stroke patients presenting with AVS with or without nystagmus [13]. In this group, objective examination findings such as gait and imbalance assessments may provide valuable diagnostic information [4, 14, 15], which is also incorporated in an abbreviated form in the STANDING algorithm [16].

The presentation with acute‐onset imbalance without nystagmus is often referred to as the acute imbalance syndrome (AIS) [1, 10, 17, 18, 19, 20] or acute truncal ataxia [11]. Despite its clinical relevance, this subgroup has rarely been studied prospectively. To address this limitation and to gain more knowledge about its clinical presentation in acutely dizzy patients presenting to the ED, we conducted a systematic review of the literature.

## 2 Methods

### 2.1 Data Sources and Searches

We searched MEDLINE and Embase for English‐language original articles, using the following components: (1) presenting symptoms including imbalance, gait disturbance, ataxia, vertigo or dizziness (2) underlying causes including stroke and acute unilateral vestibulopathy, but also other (non‐)vestibular causes (3) reporting on epidemiology, clinical presentation or differential diagnosis, (4) focusing on acute presentations. The search string was designed by a neurologist with relevant domain expertise in neuro‐otology (AAT) and is provided in Appendix 1. We limited the search to 2000–2024 (last update: December 5th, 2024) to ensure modern diagnostic techniques were applied. We also conducted a manual search of the reference lists from eligible articles and contacted corresponding authors where necessary. We did not seek to identify research abstracts from meeting proceedings or unpublished studies. Being a systematic review, no ethical approval was required. This systematic review has been registered on PROSPERO (CRD42024623068) and follows PRISMA guidelines.

### 2.2 Study Selection

Articles were screened independently by two reviewers (KS, AAT) using predefined inclusion and exclusion criteria and a structured process. Screening was performed in two stages, first at the title/abstract level and subsequently at the full‐text level. Detailed exclusion criteria are provided in Appendix 1. Full‐text screening was performed for all citations classified as “yes” or “maybe” using the same inclusion and exclusion criteria as for title/abstract screening. Disagreements during the full‐text assessment were resolved by consensus. Inter‐rater agreement for full‐text inclusion was calculated using Cohen's kappa [21]. A formal quantitative risk‐of‐bias assessment was not performed, as the included studies were heterogeneous in design and primarily descriptive in nature. For such a setting, a quantitative risk‐of‐bias assessment would not provide additional meaningful information to the qualitative assessments made.

### 2.3 Data Extraction

Predefined data were extracted from all eligible studies. Variables included study design (prospective, retrospective), country, and study period; population characteristics (sample size, age, sex, inclusion/exclusion criteria); diagnostic and clinical findings (symptoms, signs, examination results), as well as the final diagnosis. We studied both AVS/AIS patients with additional, obvious focal neurologic symptoms and those with isolated vestibular symptoms.

### 2.4 Terminology

To ensure consistency across studies that used heterogeneous wording and definitions, several umbrella terms were applied. Imbalance was used to denote any acute disturbance of stance, gait, or postural stability, including unsteadiness, disequilibrium, truncal ataxia, or gait instability—in line with the Bárány Society's definition of “postural symptoms” [22]. Gait instability was used as an umbrella term encompassing all disturbances of gait pattern, coordination, or stability while walking, regardless of the underlying mechanism or descriptive label used in the original study. Vertigo was used as an umbrella term referring to a false or distorted sensation of self‐motion of the head or body, consistent with the definition proposed by the Bárány Society [22].

In order to study the clinical spectrum of the AIS, we required patients to present without nystagmus and with acute‐onset and persistent imbalance of stance or gait and/or vertigo/dizziness. Thus, to capture AIS presentations broadly, we also included patients without reported imbalance yet vertigo or dizziness without nystagmus was described, as well as cases where the original report described imbalance or vertigo without clearly specifying which symptom predominated. By using the ICD‐11 definition of AVS (with nystagmus being a nonmandatory feature), AIS became a subcategory of AVS. Presence of either spontaneous nystagmus (SN) or gaze‐evoked nystagmus (GEN) was considered sufficient to count as “nystagmus present.” This is in accordance with the previously used definition of AVS by Kattah [5] requiring presence of either SN or GEN.

We reviewed all studies included with regards to the definition of AVS, if such a definition was provided. In all other studies, we evaluated whether the patients met the diagnostic criteria for AVS (according to ICD‐11) as reported. The number of AVS cases reported in our review always includes those with and without nystagmus presentation. The absence of nystagmus was assumed when explicitly stated, or when nystagmus was not reported in studies that provided nystagmus findings and either stated that all patients had been examined or gave reasonable grounds to assume that all had been tested.

### 2.5 Data Analysis

Populations were grouped according to the way patients were enrolled and reported. We distinguished between symptom‐based populations and diagnosis‐ or syndrome‐specific populations. These grouped populations formed the basis for the descriptive analysis of clinical characteristics and for the subsequent tabulation of AIS‐related findings presented in the results section. Whenever possible, findings from studies were merged, and a quantitative meta‐analysis across studies was performed.

## 3 Results

**FIGURE 1:**
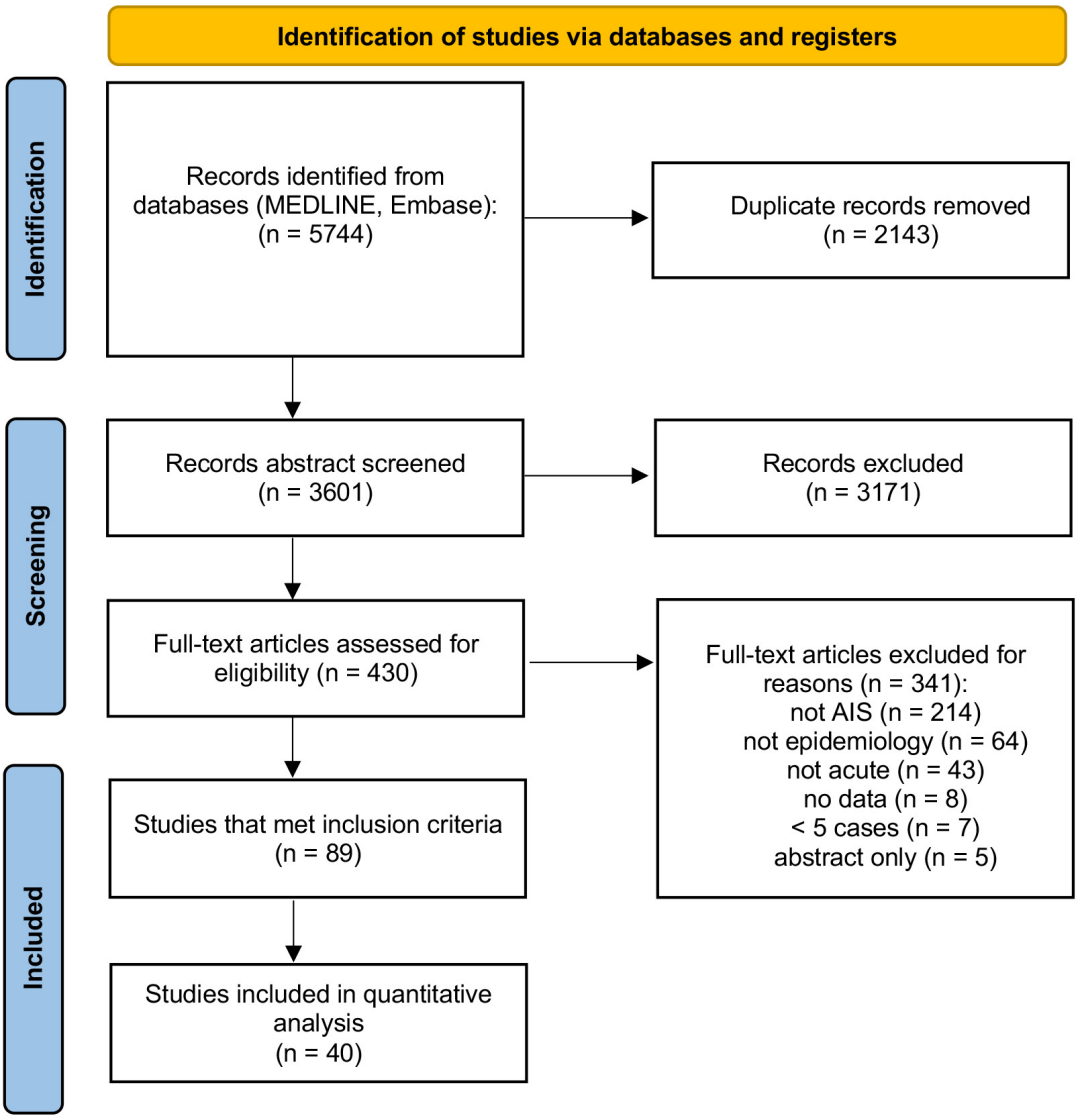
PRISMA flow chart of the literature search and article selection, adapted from [23]. MEDLINE was accessed via PubMed; Embase was accessed via embase.com. Full‐text screening was performed for all citations classified as “yes” or “maybe” using the same inclusion and exclusion criteria as for title/abstract screening. Disagreements during full‐text screening were resolved by consensus.

Our search identified 3601 unique citations, of which 3171 (88%) were excluded at the title and abstract level. After resolving disagreements at the full‐text level, 89 of 430 studies were considered eligible, representing 2.5% of all screened records (see Figure 1‐ Prisma flow chart). Inter‐rater agreement for inclusion of full‐text manuscripts was high (*κ* = 0.88, 95%–CI = 0.85–0.91). Amongst all full‐text manuscripts excluded (79.3%), the distribution of reasons for exclusion was as follows: 49.8% were not about vertigo, dizziness or imbalance, 14.9% were not about the epidemiology, frequency/differential diagnosis or clinical presentation, 10.0% did not include data about acute (< 72 h) vertigo, dizziness or imbalance, 1.9% contained no original data, 1.6% had < 5 subjects studied, and 1.2% were conference abstracts only.

### 3.1 Study Characteristics

From the 89 eligible studies, we identified 40 studies that reported on patients presenting with acute vertigo/dizziness or imbalance and that provided information about the presence/absence of nystagmus for the entire cohort. These 40 studies formed the basis of our descriptive synthesis/meta‐analysis and comprised 11,371 patients overall (see Appendix 2: Table A1 for details). Nineteen studies were prospective, 21 were retrospective. Whereas inclusion criteria were symptom‐based in 13 studies, 21 studies required a specific diagnosis (ischemic stroke in all but one study) for inclusion and the remaining 6 studies used syndrome‐based inclusion criteria (AVS) (see Table 1).

**TABLE 1:** Epidemiologic key findings.

	Studies (*n*)	Patients (*n*)
Gender		
Female	30	5390
Male	30	4878
Not reported	10	1463
All	40	11,731
Study type		
Prospective observational	19	7915
Retrospective cross‐sectional	21	3816
Patient enrolment		
Consecutive	20	8493
Likely consecutive	3	806
Nonconsecutive	14	1142
Unclear	3	1290
Number of study sites		
Monocentric	33	8731
Multicentric	7	2986
Patient population		
Acute vertigo/dizziness/imbalance presenting to ED (symptom‐based)	13	9339
Strokes presenting with acute vertigo/dizziness/imbalance (diagnosis‐based)	9	253
AVS/AIS presenting to ED (syndrome‐based)	6	1700
Strokes presenting with AVS/AIS to ED (diagnosis‐based)	12	439
Imaging		
MRI‐DWI all	25	1893
MRI or CT in all	7	1633
MRI or CT in some	7	8192
CT in all	1	13

Abbreviations: AIS, acute imbalance syndrome; AVS, acute vestibular syndrome; CT, computed tomography; DWI, diffusion‐weighted imaging; ED, emergency department; MRI, magnetic resonance imaging.

Amongst those studies, diagnostic criteria for AVS/AIS were met in 18, reporting on a total of 2730 patients. MR‐imaging was obtained in all patients in 25 studies (*n* = 1893 patients), whereas in 7 studies (*n* = 1633 patients) either MRI‐DWI or CT was retrieved. Noteworthy, in 7 studies (*n* = 8192 patients) brain imaging was obtained in part of the patients only and in one study CT was used for identifying patients with intracranial hemorrhage.

### 3.2 The Range of Nystagmus‐Prevalence Amongst Symptom‐Based Studies

Patient selection amongst studies that included participants based on presenting symptoms (i.e., acute vertigo, dizziness or imbalance) varied substantially. While some studies included all acutely dizzy patients without restrictions, others focused on isolated presentations (i.e., without obvious focal neurologic signs), required retrieval of MRI‐DWI and/or excluded patients with other clearly identifiable causes of dizziness (see Table 2). Overall, the range of percentages of patients that demonstrated nystagmus was broad (8.9% to 95.7%). Focusing on those patients with central diagnoses (and including also those studies that reported on central causes of acute vertigo, dizziness or imbalance), the fraction of patients with nystagmus was between 12.2% and 100%. In those studies that included also acutely dizzy patients with noncentral causes or nonstroke causes, again a broad range of nystagmus fractions was seen (8.7% to 100% and 12.8% to 78.8%, respectively).

**TABLE 2:** Frequency of nystagmus in symptom‐based studies.

Authors/Publication year	ED patient population	Study sample (*n*)	Nystagmus amongst all dizzy patients			
			All (%)	Central origin (%)	Noncentral origin (%)	Nonstroke origin (%)
**Studies reporting on both central and noncentral causes of acute vertigo, dizziness and imbalance**						
Ammar et al. 2017 [24]	Acute dizziness or imbalance	307	14.01%	28.57%	13.67%	NA
Chase et al. 2014 [26]	Isolated acute dizziness (i.e., no focal neurologic findings), no other clearly identifiable causes of dizziness	426	21.83%	38.46%^a^	NA	21.3%
Chase et al. 2012 [27]	Acute vertigo and having received brain MRI	131	21.37%	33.33%^a^	NA	20.2%
Choi et al. 2017 [30]	Acute vertigo or dizziness lasting < 1 day, excluding those with positional vertigo, other medical conditions or preexisting neurologic disorders.	23	95.65%	87.50%	100.00%	NA
Jo et al. 2019 [36]	Isolated acute vertigo or dizziness	468	45.51%	42.11%	45.81%	NA
Kerber et al. 2015 [39]	Nonisolated acute dizziness (i.e., with examination findings)	272	73.90%	82.76%^a^	NA	72.8%
Kmetonyova et al. 2023 [41]	Acute dizziness and gait instability (no head trauma < 48 h)	119	69.75%	51.28%^a^	NA	78.8%
Li et al. 2024 [45]	Isolated vertigo/dizziness, other medical conditions excluded	109	38.53%	44.00%	36.90%	NA
Navi et al. 2012 [50]	Acute dizziness	907	8.93%	12.24%	8.74%	NA
Ohle et al. 2025 [55]	Acute vertigo, dizziness or imbalance	2078	9.19%	12.61%	9.00%	NA
Sandlund et al. 2019 [56]	Acute vertigo or dizziness	3613	10.16%	NA	NA	NA
Vanni et al. 2017 [58]	Acute vertigo or unsteadiness, other medical causes excluded	352	78.41%	50.00%	75.64%	NA
Zuo et al. 2018 [60]	Acute vertigo or dizziness, only those with NIHSS = 0 and having received brain MRI/CT	273	12.45%	10.87%^a^	NA	12.8%
**Studies reporting on central causes of vertigo, dizziness and imbalance only**						
Braun et al. 2011 [25]	Acute dizziness or imbalance initially diagnosed as peripheral, but with MRI‐positive lesions	12		75.0%		
Deluca et al. 2011 [32]	Acute imbalance in cerebellar stroke	92		65.2%		
Eguchi et al. 2019 [34]	Acute vertigo/dizziness in ACS	8		12.5%		
Honda et al. 2014 [35]	Acute imbalance in MRI‐confirmed stroke	41		31.7%		
Kerber et al. 2012 [38]	Acute dizziness and NIHSS < 2 in intracranial hemorrhage	13		15.4%		
Lee and Baloh 2005 [42]	Sudden deafness and vertigo in PCS	28		100.0%		
Lee et al. 2013 [43]	Acute vertigo or dizziness in SCA stroke	31		35.5%		
Mandge et al. 2020 [47]	Acute vertigo or dizziness in stroke	14		42.9%		
Yang et al. 2019 [59]	Acute vertigo in PICA stroke	14		85.7%		

Abbreviations: ACS, anterior circulation stroke; CT, computed tomography; ED, emergency department; MRI, magnetic resonance imaging; NA, not available; NIHSS, National institutes of health stroke scale; NR, not reported; PCS, posterior circulation stroke; PICA, posterior‐inferior cerebellar artery; SCA, superior cerebellar artery.

^a^ All central cases were ischemic strokes (comparison with nonstroke cases).

When pooling all symptom‐based studies that included both central and noncentral causes, nystagmus was reported in 32.1% (134/417) of all central causes (including strokes) and in 19.5% (772/3966) of all noncentral causes. For those studies that included only acutely dizzy patients with central causes and did not provide sufficient information to allow for a syndromal classification, 56.3% (142/252) presented with nystagmus. Noteworthy, no individual patient data were available linking nystagmus presence/absence to specific diagnoses or vascular territory affected in the case of stroke.

### 3.3 The Range of AIS‐Prevalence Amongst Syndrome (AVS)‐Based Studies

In Table 3 all studies with a syndrome‐based inclusion are shown. Amongst those six studies that included central and noncentral AVS‐cases, four studies had more restrictive inclusion criteria, either excluding those cases with other clear medical causes of dizziness or focusing on posterior‐circulation strokes (PCS) and acute unilateral vestibulopathy (AUVP). Amongst those 12 studies that included only cases with central AVS, 11 were focusing on PCS, often including only strokes within a specific vascular territory or with predefined anatomical structures affected (such as the cerebellar nodulus), selected clinical presentations (e.g., NIHSS < 5 points) or with initially negative MRI‐DWI. Fractions of AIS‐cases ranged between 7.4% and 60.9% in combined (central/noncentral) cohorts, whereas in studies reporting only on central AVS, the fraction of AIS cases was between 7.7% and 77.6%, depending on the patient selection (see Table 3). A single study assessed the fraction of AIS‐cases amongst all acutely dizzy patients presenting to the ED, reporting a value of 30.7% [31].

**TABLE 3:** Frequency of AIS in AVS‐based studies.

Studies reporting on both central and noncentral AVS cases							
Authors/Publication year	All ED dizzy patients	Patient selection	AVS		AIS		
			Central cases	Noncentral cases	Amongst all AVS (%)	Amongst all central AVS (%)	Amongst all peripheral AVS (%)
Carmona et al. 2023 [11]	NR	All AVS cases	64	31	14.7%	21.9%	0.0%
Comolli et al. 2023 [31]	1535	All AVS cases	192	223	60.9%	52.6%	35.0%
Liu et al. 2024 [46]	NR	AVS (PCS and AUVP only)	52	69	7.4%	11.5%	4.3%
Machner et al. 2020 [19]	NR	AVS requiring admission to neurology ward (those with dizziness due to a clear medical cause or discharged from ED were excluded)	69	173	53.3%	62.3%	49.7%
Nham et al. 2023 [51]	NR	AVS (PCS and AUVP only)	101	104	21.5%	43.6%	0.0%
Nham et al. 2023 [52]	NR	AVS (PCS and AUVP only)	128	134	26.0%	53.1%	0.0%
All	NR		606	734	43.3%	45.5%	22.8%

	All stroke cases	Patient selection	Central AVS			AIS amongst all central AVS (%)	
Choi et al. 2014 [28]	132	AVS in PCS (no obvious focal neurologic signs, altered mental status)	34	NA	NA	67.6%	NA
Choi et al. 2018 [29]	1846	AVS in PCS with early negative brain MRI‐DWI	35	NA	NA	54.3%	NA
Dieterich et al. 2018 [33]	158	AVS in unilateral midbrain stroke	23	NA	NA	39.1%	NA
Kattah et al. 2023 [37]	NR	AVS in acute medullary stroke	13	NA	NA	7.7%	NA
Kim et al. 2019 [40]	NR	AVS in middle cerebellar peduncle stroke	23	NA	NA	8.7%	NA
Lee et al. 2023 [44]	NR	AVS in isolated (hemi)nodular stroke	5	NA	NA	60.0%	NA
Moon et al. 2009 [48]	NR	AVS in isolated nodular stroke	8	NA	NA	12.5%	NA
Morita et al. 2011 [49]	NR	AVS in cerebellar stroke but early negative brain MRI‐DWI	8	NA	NA	25.0%	NA
Nikles et al. 2024 [13]	NR	AVS in MRI‐confirmed stroke	115	NA	NA	48.7%	NA
Ogawa et al. 2016 [54]	NR	Isolated AVS in cerebellar stroke/hemorrhage initially considered of peripheral‐origin	11	NA	NA	54.5%	NA
Saro‐Buendia et al. 2023 [57]	NR	AVS in PCS confirmed by MRI‐DWI	85	NA	NA	77.6%	NA
Shi et al. 2022 [20]	NR	AVS in MRI‐confirmed stroke and NIHSS < 5	79	NA	NA	62.0%	NA
All	2136		439	NA	NA	54.0%	NA

Abbreviations: AIS, acute imbalance syndrome; AUVP, acute unilateral vestibulopathy; AVS, acute vestibular syndrome; DWI, diffusion‐weighted imaging; ED, emergency department; MRI, magnetic resonance imaging; NA, not available; NIHSS, National institutes of health stroke scale; NR, not reported; PCS, posterior circulation stroke.

### 3.4 Patient Characteristics Across All Studies and Patient Groups

Whereas all patients presented with either vertigo, dizziness, or imbalance, information on the presence/absence of imbalance was lacking in 66.3% (7779/11731) of patients. When assessed, imbalance was reportedly present in 76.1% of cases (including both central/nonstroke/noncentral causes) (see Table 4). Whereas 76.7% of all patients were included based on presenting symptoms, 23.3% had received a syndromal diagnosis of either AVS with nystagmus (12.3%) or AIS (11.0%). The fraction of patients with reported nystagmus was 25.3% when pooling all patients irrespective of their underlying diagnosis and presenting symptoms. However, there was substantial variation across subgroups. Specifically, the fraction of patients with nystagmus was highest for the subgroup with central causes (47.1%), whereas smaller fractions were seen for the subgroups with nonstroke causes (33.8%) and with noncentral causes (27.3%), respectively.

**TABLE 4:** Presenting symptoms, signs and diagnoses reported across all studies (*n* = 40) included.

	Central causes (including strokes)	Noncentral causes	Nonstroke causes (peripheral/central)	No subgroups/Diagnoses	All
**Reported symptoms**					
Vertigo, dizziness or imbalance	1717 (14.6%)	4913 (41.9%)	1128 (9.6%)	3973 (33.9%)	11,731 (100%)
Imbalance					
Present	824 (48.0%)	1205 (24.5%)	617 (54.7%)	360 (9.1%)	3006 (25.6%)
Absent	255 (14.8%)	180 (3.7%)	511 (45.3%)	0 (0%)	946 (8.1%)
Not reported	638 (37.2%)	3528 (71.8%)	0 (0%)	3613 (90.9%)	7779 (66.3%)
**Presenting symptom/syndrome**					
AVS (with/without nystagmus)	1045 (60.9%)	734 (14.9%)	0 (0%)	951 (23.9%)	2730 (23.3%)
AVS with nystagmus	532 (50.9%)	567 (77.2%)	0 (0%)	339 (35.6%)	1438 (52.7%)
AIS (AVS without nystagmus)	513 (49.1%)	167 (22.8%)	0 (0%)	612 (64.4%)	1292 (47.3%)
Symptom based only	672 (39.1%)	4179 (85.1%)	1128 (100.0%)	3022 (76.1%)	9001 (76.7%)
**Nystagmus**					
Present	808 (47.1%)	1339 (27.3%)	381 (33.8%)	434 (10.9%)	2962 (25.3%)
Absent	906 (52.8%)	3361 (68.4%)	701 (62.1%)	3539 (89.1%)	8507 (72.5%)
Not reported	3 (0.1%)	213 (4.3%)	46 (4.1%)	0 (0%)	262 (2.2%)
**Diagnosis**					
All ischemic strokes^a^	1529 (89.1%)	NR	NR	NR	1529 (13.0%)
PICA	328 (21.1%)	NR	NR	NR	328 (2.3%)
AICA	81 (5.2%)	NR	NR	NR	81 (0.7%)
SCA	80 (5.1%)	NR	NR	NR	80 (0.6%)
Other lesion locations^b^	404 (25.9%)	NR	NR	NR	404 (2.8%)
Nonspecified	664 (42.6%)	NR	NR	NR	664 (6.6%)
Intracranial hemorrhage	24 (1.4%)	NR	NR	NR	24 (0.2%)
Tumor	41 (2.4%)	NR	NR	NR	41 (0.3%)
Demyelinating disease	8 (0.5%)	NR	NR	NR	8 (0.1%)
Other central disorders^c^	108 (6.3%)	NR	NR	NR	108 (0.9%)
Not reported	7 (0.4%)	NR	NR	NR	7 (0.1%)
**All central disorders**	**1717 (100%)**	NR	NR	NR	**1717 (14.6%)**
Acute unilateral vestibulopathy	NR	610 (12.4%)	NR	NR	610 (5.2%)
BPPV	NR	719 (14.6%)	NR	NR	719 (6.1%)
Other noncentral disorders^d^	NR	1440 (29.3%)	NR	NR	1440 (12.3%)
Nonspecified noncentral disorders	NR	2144 (43.6%)	NR	NR	2144 (18.3%)
**All noncentral disorders**	NR	**4913 (100%)**	NR	NR	**4913 (41.9%)**
**All nonstroke diagnoses**	NR	NR	**1128 (100%)**	NR	**1128 (9.6%)**
**No diagnosis provided**	NR	NR	NR	**3973 (100%)**	**3973 (33.9%)**

Abbreviations: ACA, anterior cerebral artery; AICA, anterior inferior cerebellar artery; AIS, acute imbalance syndrome; AVS, acute vestibular syndrome; BA, basilar artery; BPPV, benign paroxysmal positional vertigo; MCA, middle cerebral artery; PCA, posterior cerebral artery; PICA, posterior‐inferior cerebellar artery; PPPD, persistent postural‐perceptual dizziness; SCA, superior cerebellar artery; TIA, transient ischemic attack; VA = vertebral artery.

^a^ In one study, involvement of multiple vascular territories in single patients were reported separately, thus, the number of stroke lesions (*n* = 143) exceeded the number of patients (*n* = 115) [13]. For specific stroke locations there the total number (*n* = 1557) is larger than for “all ischemic strokes.”

^b^ This includes both strokes with no vascular territory reported and strokes in other vascular territories: BA strokes (*n* = 48), VA strokes (*n* = 15), PCA strokes (*n* = 36), MCA strokes (*n* = 17), ACA strokes (*n* = 7), strokes in multiple territories (*n* = 60, including PICA + AICA [*n* = 3], SCA + PICA [*n* = 4], PICA + AICA + SCA [*n* = 2]), cerebellar strokes (*n* = 53), brainstem strokes (*n* = 133), combined brainstem‐cerebellar strokes (*n* = 19), thalamic strokes (*n* = 3), supratentorial strokes (*n* = 10) involving the centrum semiovale (*n* = 1) or not being further specified (*n* = 9), and combined supratentorial and infratentorial strokes (*n* = 3).

^c^ This includes TIAs (*n* = 36), vascular malformations (aneurysms [*n* = 7], venous anomalies [*n* = 4], cavernous hemangioma [*n* = 2]), vestibular migraine (*n* = 11), cerebral trauma (*n* = 10), neurodegenerative disorders (*n* = 9), PPPD (*n* = 8), mega cisterna magna (*n* = 2), miscellaneous (*n* = 5, single cases of amyloid angiopathy, arachnoid granulation, basilar artery dissection, subdural hygroma, hydrocephalus), seizures (*n* = 4), Wernicke encephalopathy (*n* = 3), drug intoxication with anti‐seizure medications (*n* = 2), and unclear cases (*n* = 5).

^d^ This includes peripheral vestibular disorders (Menière's disease [*n* = 29], sudden hearing loss [*n* = 2], acoustic neuroma [*n* = 2], Ramsey Hunt syndrome [*n* = 2], labyrinthine ischemia [*n* = 7], labyrinthitis [*n* = 7], unspecified peripheral disorders [*n* = 185]), other neurologic disorders merged with noncentral causes in two studies (migraine [*n* = 56], concussion [*n* = 11], vertebrobasilar insufficiency [*n* = 19] [50, 58]), cardiac disorders (cardiac arrhythmia [*n* = 22], hypertensive emergency [10], acute coronary syndrome [*n* = 2], heart failure [*n* = 1], heart disease not further specified [*n* = 9]), psychiatric disorders [*n* = 22], internal medicine related disorders (orthostasis [*n* = 168], syncope [*n* = 20], drug‐related side effects [*n* = 54], systemic infections [*n* = 40], electrolyte disorders [*n* = 14], anemia [*n* = 10], hypoglycemia [4], other metabolic disorders [6], unspecified dizziness [*n* = 200], other not specified causes [*n* = 538]).

The largest fraction of patients had noncentral diagnoses (41.9%), whereas 14.6% were diagnosed with central diagnoses and another 9.6% were classified as having diagnoses other than stroke (including other central causes). No diagnosis was provided in 33.9% of cases. Within the central disorders group, ischemic strokes made up 89.1% of all cases, with posterior‐inferior cerebellar artery (PICA), anterior‐inferior cerebellar artery (AICA), and superior cerebellar artery (SCA) strokes being most frequently found (see Table 4). In the subgroup of noncentral disorders, benign paroxysmal positional vertigo (BPPV, 14.6%) and AUVP (12.4%) were the most common disorders identified. Another 29.3% of cases had various noncentral causes (with unspecified peripheral disorders [*n* = 185], unspecified dizziness [*n* = 200], and orthostatic dysregulation [*n* = 168] being most frequently reported), and in 43.6% of cases no specific diagnosis was provided. In the subgroup of nonstroke causes, none of the five studies provided further information on specific diagnoses [26, 27, 39, 41, 60].

### 3.5 AVS Vs. AIS Across Studies and Subgroups

Overall, 47.3% (1292/2730) of all AVS cases (with or without nystagmus) presented without nystagmus, thus fitting the criteria of an AIS. For the different subgroups, the fraction of AIS among all AVS varied substantially as shown in Table 4. Specifically, the fraction of AIS cases was larger in patients with central causes compared to those with noncentral causes (49.1% vs. 22.8%).

The distribution of specific diagnoses in studies reporting on AVS with nystagmus versus AIS and its breakdown for the different subgroups is shown in Table 5 (for central causes) and in Table 6 (for noncentral causes). In two studies no breakdown for AVS with nystagmus/AIS in central versus noncentral subgroups was provided for certain subgroups (other, nonspecified diagnoses and unclear diagnoses). Therefore, 951 patients from these two studies were excluded from this analysis [31, 56]. In addition, two studies did not provide the breakdown for stroke location for AVS with nystagmus versus AIS [46, 52], thus, the 180 stroke cases from those studies were re‐classified as “not specified” for this sub analysis.

**TABLE 5:** Distribution of diagnoses amongst central AVS and AIS cases.

	AVS with nystagmus (%)	AIS (%)	AVS all (with/without nystagmus) (% all causes)
**Central disorders**			
Ischemic strokes			
PICA	111 (59.7%)	75 (40.3%)	186 (17.3%)
AICA	39 (90.7%)	4 (9.3%)	43 (4.0%)
SCA	10 (43.5%)	13 (56.5%)	23 (2.1%)
PCA	8 (38.1%)	13 (61.9%)	21 (2.0%)
Multiple vascular territories	14^a^ (56.0%)	11^b^ (44.0%)	25 (2.3%)
Other lesion locations	71^c^ (48.3%)	76^d^ (51.7%)	147 (13.7%)
Nonspecified	256 (47.1%)	288 (52.9%)	544 (50.7%)
**All ischemic strokes** ^e^	**492 (51.2%)**	**469 (48.8%)**	**961 (92.0%)**
Intracranial hemorrhage	1 (100%)	0 (0%)	1 (0.1%)
Tumor	3 (21.4%)	11 (78.6%)	14 (1.3%)
Demyelinating disease	3 (75.0%)	1 (25.0%)	4 (0.4%)
Vestibular migraine	10 (90.9%)	1 (9.1%)	11 (1.1%)
TIA	8 (47.1%)	9 (52.9%)	17 (1.6%)
Brain trauma	0 (0%)	9 (100%)	9 (0.9%)
PPPD	1 (12.5%)	7 (87.5%)	8 (0.8%)
Wernicke encephalopathy	3 (100%)	0 (0%)	3 (0.3%)
Antiseizure medication induced	2 (100%)	0 (0%)	2 (0.2%)
Miscellaneous	4 (50.0%)	4 (50.0%)	8 (0.8%)
Not reported	2 (28.6%)	5 (71.4%)	7 (0.7%)
**All central disorders**	**529 (50.6%)**	**516 (49.4%)**	**1045 (100%)**

Abbreviations: ACA, anterior cerebral artery; AICA, anterior inferior cerebellar artery; AIS, acute imbalance syndrome; AVS, acute vestibular syndrome; BA, basilar artery; MCA, middle cerebral artery; PCA, posterior cerebral artery; PICA, posterior‐inferior cerebellar artery; PPPD, persistent postural‐perceptual dizziness; SCA, superior cerebellar artery; TIA, transient ischemic attack; VA, vertebral artery.

^a^ Multiple vascular territories include PICA + SCA (*n* = 3), PICA + AICA (*n* = 2), nonspecified (*n* = 9).

^b^ Multiple vascular territories include PICA + SCA + AICA (*n* = 1), PICA + SCA (*n* = 1), nonspecified (*n* = 9).

^c^ Other lesion locations include brainstem lesions (basilar perforators [*n* = 9], midbrain perforators [*n* = 17], nonspecified [*n* = 11]), combined brainstem and cerebellar lesions (*n* = 2), cerebellar lesions (*n* = 3), BA (*n* = 14), VA (*n* = 9), MCA (*n* = 5), ACA (*n* = 1).

^d^ Other lesion locations include brainstem lesions (basilar perforators [*n* = 9], midbrain perforators [*n* = 14], not further specified [*n* = 14]), cerebellar lesions not further specified [*n* = 3], combined brainstem and cerebellar lesions [*n* = 2], MCA strokes (*n* = 10), ACA (*n* = 3), VA strokes (*n* = 4), BA strokes (*n* = 14), thalamic strokes (*n* = 3).

^e^ In one study, involvement of multiple vascular territories in single patients were reported separately, thus, the number of stroke lesions (*n* = 143) exceeded the number of patients (*n* = 115) [13]. For specific stroke locations therefore the total number (*n* = 989) is larger than for “all ischemic strokes.”

**TABLE 6:** distribution of diagnoses amongst noncentral AVS and AIS cases.

	AVS with nystagmus	AIS	AVS all (with/without nystagmus)
**Peripheral‐vestibular disorders**			
AUVP	453 (97.6%)	11 (2.4%)	464 (63.2%)
Labyrinthine ischemia	7 (100%)	0 (0%)	7 (1.0%)
Ramsey hunt syndrome	2 (100%)	0 (0%)	2 (0.3%)
Labyrinthitis	7 (100%)	0 (0%)	7 (1.0%)
Acoustic neuroma	1 (50%)	1 (50%)	2 (0.3%)
BPPV	1 (50%)	1 (50%)	2 (0.3%)
Menière's disease	1 (100%)	0 (0%)	1 (0.1%)
**All peripheral‐vestibular disorders**	**472 (97.3%)**	**13 (2.7%)**	**485 (66.1%)**
Other noncentral disorders			
Dysautonomia	0 (0%)	47 (100%)	47 (6.4%)
Medication side effect	0 (0%)	8 (100%)	8 (1.1%)
Hearth disease	1 (11.1%)	8 (88.9%)	9 (1.2%)
Infectious disease	6 (100%)	0 (0%)	6 (0.8%)
Metabolic	1 (16.7%)	5 (83.3%)	6 (0.8%)
Nonspecified	87 (50.3%)	86 (49.7%)	173 (23.6%)
**All other noncentral disorders**	**95 (38.2%)**	**154 (61.8%)**	**249 (33.9%)**
**All noncentral disorders**	**567 (77.2%)**	**167 (22.8%)**	**734 (100%)**

Abbreviations: AIS, acute imbalance syndrome; AUVP, acute unilateral vestibulopathy; AVS, acute vestibular syndrome; BPPV, benign paroxysmal positional vertigo.

Whereas overall ischemic strokes presented as AVS with nystagmus or as AIS at similar frequency (51.2% vs. 48.8%), the vascular territories affected and the anatomical structures involved had a major impact on the clinical presentation. The highest rates of AVS with nystagmus were seen in AICA‐strokes, reaching 90.7%. In PICA‐strokes, 59.7% of cases presented as AVS with nystagmus, whereas for SCA‐strokes (43.5%) and PCA‐strokes (38.1%) this was the case only in a minority of patients. For the remaining subcategories (multiple vascular territories, other specified lesion locations, nonspecified lesion locations), the distribution between AVS with nystagmus and AIS cases was fairly even, ranging between 47.1% to 56.0% for the fractions of AVS with nystagmus. For other, less frequently reported central causes, again varying distributions (AVS with nystagmus vs. AIS) were seen, with a majority of AVS with nystagmus cases for vestibular migraine (90.9%), Wernicke encephalopathy (100%) and intoxication due to antiseizure medications (100%). In contrast, AIS cases dominated in the case of persistent postural perceptual dizziness (87.5%) and traumatic brain injury (100%).

Amongst the noncentral causes, overall patients presenting with AVS with nystagmus were in the majority, representing 77.2% of the total. When focusing on peripheral‐vestibular disorders, the fraction of AVS with nystagmus was even higher, reaching 97.3%, mostly due to the high prevalence of AUVP (97.6%, 453/464) presenting as AVS with nystagmus. In contrast, amongst other noncentral causes, AIS presentations were much more frequent, reaching 100% for dysautonomia and other medication side effects, 88.9% for heart disease, and 83.3% for metabolic disorders.

## 4 Discussion

The spectrum of clinical presentations and underlying diagnoses in acutely dizzy patients presenting to the ED is very broad. The diagnostic workup of these patients is often challenging and prone to diagnostic errors [61]. Presence of subtle ocular motor findings only and imprecision in reported symptoms by the patients are potential confounders. Therefore, structured history taking and a clear distinction between different vestibular syndromes is essential in the diagnostic workup. While the TiTrATE approach provides such an algorithm [62], the diagnostic approach must be adapted to the patient's clinical presentation. The diagnostic accuracy of bedside testing as applied by the HINTS or its extension (HINTS “+”) which includes new‐onset unilateral hearing‐loss as a fourth sign [15] for identifying central causes of acute vestibular symptoms is excellent when applied in those patients presenting with nystagmus [6]. However, its diagnostic value is clearly lower when applied more broadly (including acutely dizzy patients without nystagmus) [9, 39]. Therefore, in the patient with acute prolonged vertigo and dizziness (accompanied by imbalance, nausea/vomiting and motion intolerance), the assessment of nystagmus is critical. Whereas the most up‐to‐date AVS definition (according to ICD‐11 [8]) considers nystagmus a nonmandatory feature to be more inclusive, a further distinction of subphenotypes is needed to appropriately select the most appropriate bedside‐testing algorithm. Therefore, advancing our knowledge of the clinical presentation and underlying causes of the AIS, that is, of those patients that present with an AVS without nystagmus, is critical. In the systematic review performed, we identified substantial differences in the distribution of underlying disorders in AVS with nystagmus versus AIS, emphasizing the need to adapt the diagnostic work‐up based on the presence/absence of nystagmus in acutely dizzy patients.

### 4.1 Frequency of AVS With Nystagmus and AIS Amongst Acutely Dizzy Patients

Very few studies provided information on the prevalence of AVS (with/without nystagmus) amongst all acutely dizzy patients presenting to the ED and values retrieved varied substantially, reaching 50.5% in one study [31] and only 16.4% in another study [56]. Likely, these discrepancies are related to training in applying the AVS criteria (done primarily by a medical student in the study by Sandlund and by trained neuro‐otology fellows in the study by Comolli), study design (prospective data collection vs. retrospective screening of medical records), the timing of the assessment (with missing AVS cases when done before 24 h symptom duration is reached) and the granularity of patient reported symptoms retrieved [31]. Noteworthy, amongst such relatively unselected acutely dizzy patient cohorts, AIS was more frequent than AVS with nystagmus. This was true both for the study by Comolli (30.7% vs. 19.7% [31]) and in the study by Sandlund (8.8% vs. 7.5% [56]). These findings are similar to those reported in another study that was not part of our systematic review, with a fraction of 19.2% of all acutely dizzy ED‐patients presenting as AVS, with the fraction of AIS cases being larger than that of AVS with nystagmus cases (60.5% vs. 39.5%) [63].

A further breakdown was identified in a single study only. Specifically amongst all acutely dizzy patient, the subset of central AVS cases reached 12.5% with a slight majority of AIS over AVS with nystagmus cases (6.6% vs. 5.9%), whereas for peripheral AVS (14.% in total) those cases with nystagmus were more frequent than the AIS cases (9.4% vs. 5.1%) [31]. Thus, an AVS presentation (including AIS) is found in a significant fraction of acutely dizzy patients, emphasizing the importance of a syndromal vestibular classification of these patients according to the international classification of vestibular disorders (ICVD) [22].

### 4.2 Patients Presenting With AVS With Nystagmus or AIS Have Distinct Underlying Disorders

When pooling the data from all AVS‐studies, central causes were dominated by ischemic stroke (representing 92% of all these cases) and AUVP represented the majority of noncentral causes (63.2%). In our meta‐analysis, we identified a fraction of AIS amongst all ischemic stroke cases (with anterior circulation involvement in 22/961 patients) of 48.8% and 49.4% for all central disorders, respectively. Thereby, the affected vascular territory had a major impact on the clinical presentation. Compared to AVS with nystagmus, an AIS presentation was rarely seen in AICA strokes (9% only), and less frequent in PICA strokes (40%), while it was more prevalent in SCA strokes (56%) and PCA strokes (62%). This distribution may be explained by a predominant affection of vestibular networks in the AICA/medial PICA territory in AVS with nystagmus and nonvestibular postural networks in SCA/PCA territory (including the superior cerebellar vermis, midbrain, thalamus) in AIS. For PICA strokes, different patterns were previously reported depending on the branch(es) affected. Specifically, it has been proposed that lateral‐branch PICA strokes (supporting the inferior lateral portion of the cerebellar hemisphere) rarely present with nystagmus [11, 64]. In contrast, nystagmus was more frequently seen in medial‐branch PICA strokes [11]. Data was insufficient to further assess this observation in our meta‐analysis, though.

When focusing on anatomical regions, strokes restricted to the lateral medulla [37] or the middle cerebellar peduncles [40] rarely presented as AIS in small case series, whereas data were more variable for isolated (hemi)nodular strokes with AIS rates of 12.5% [48] and 60% [44], respectively. The relatively high fraction of AIS cases in SCA strokes of 56% is also reflected in the low rate of nystagmus in SCA strokes of 35% reported in another study that did not meet diagnostic criteria for AVS [43]. For anterior circulation strokes (ACS), vestibular symptoms may be identified in a minority of cases (with fractions of 1% [34] and 3.7% [65], respectively, in two studies). If present, the majority of AVS cases (68%) presented as AIS in one study [31]. Likewise, in another study focusing on supratentorial strokes presenting with vestibular symptoms (but not providing a classification into vestibular syndromes), only one out of eight cases presented with nystagmus [34]. Noteworthy, part of the cases with vestibular symptoms linked to ACS may have additional ischemic lesions in the posterior circulation based on focal neurologic deficits identified, but may present with negative initial MR imaging. Repeated MRI‐DWI may be considered in these cases as discussed by Nikles and colleagues [13].

Overall, absence of nystagmus in central AVS is frequent. With > 90% of central AIS cases in our meta‐analysis being linked to ischemic stroke, this should trigger a stroke workup as well in those patients. Interestingly, focusing on those studies reporting on isolated central AVS presentations, AIS cases were in the majority, reaching 55% [54, 66] and 68% [28], respectively. This underlines the impact of isolated presentations and absence of nystagmus in central AVS and the importance of selecting testing algorithms appropriately, as discussed further below.

Not surprisingly, in those studies focusing on posterior circulation stroke (PCS) and acute unilateral vestibulopathy (AUVP), virtually all peripheral AVS cases belonged to the AVS with nystagmus group [46, 51, 52] as presence of nystagmus is a feature of AUVP by definition [67]. Thus, absence of nystagmus was a strong predictor for central (ischemic stroke) causes in these studies. When broadening the inclusion criteria, the fraction of noncentral causes presenting as AIS increased to 22.8% and reached 35% in a study with relatively unselected AVS patients [31]. Nonetheless, in this study, an AIS presentation made a central cause 46% more likely than an AVS with nystagmus presentation (56% vs. 39% central causes for AIS vs. AVS), emphasizing a disproportionately high risk of central pathology in AIS. The risk of central AIS may further increase with a higher ABCD^2^ score ≥ 4 [19]. Thus, a stroke workup in AIS cases is at least as much needed as in AVS with nystagmus presentations. In contrast, vestibular migraine (1 out of 11 cases) and Wernicke Encephalopathy (0/3 cases) rarely presented as AIS, albeit with small numbers and data mainly from a single study [11].

Data on underlying causes in noncentral AIS presentations was very limited, with only a single study reporting specific diagnoses in 45% of cases [31] and a second study providing no details at all [19]. In this single study, the most frequent diagnoses reported were dysautonomia (67%), medication side effects (11%), and heart disease (11%), providing some guidance in the diagnostic workup of noncentral AIS cases. However, further research on the differential diagnosis of noncentral AIS presentations is urgently needed.

### 4.3 The Impact of Identifying Spontaneous or Gaze‐Evoked Nystagmus in Acutely Dizzy Patients

A substantial number of studies identified in our systematic review of the literature reported on nystagmus, but fell short to provide sufficient detail to allow a syndromal vestibular classification of patients included. Not surprisingly, spontaneous or gaze‐evoked nystagmus amongst acutely dizzy patients presenting to the ED was reported with substantially varying frequency, ranging from fractions as low as 9% to fractions exceeding 95% as shown in Table 2. These differences are likely reflecting patient selection based on presenting symptoms, required diagnostic standards and symptom duration, but may also be linked to training in recognizing/classifying nystagmus patterns and equipment used (e.g., assessing nystagmus only with fixation vs. both with and without fixation). Noteworthy, in those with isolated acute dizziness or vertigo, nystagmus was present at rates of 22% to 44% [26, 36, 45].

We found that in these studies, nystagmus was found at a very similar frequency both for central (32.1%) and noncentral (35.2%) causes, allowing no prediction for central versus peripheral based on the presence/absence of nystagmus. While no information on the beating pattern of spontaneous nystagmus was available from the studies included in this meta‐analysis, it is well‐known that specific patterns may be predictive for a central cause. This is true for purely vertical, purely torsional and vertical‐torsional spontaneous nystagmus [12]. Thus, in cases with nystagmus being present, an analysis of beating direction should always be performed.

When focusing on cases with confirmed central causes of acute dizziness, vertigo or imbalance (all ischemic strokes except for intracranial hemorrhage in a single study), again very variably fractions of nystagmus were observed (range = 13%–100%), likely due to the vascular territory being affected. Not surprisingly, those with combined acute audio‐vestibular losses (linked to AICA‐strokes in most cases [42]) all presented with nystagmus, whereas rates of nystagmus‐positive cases were substantially lower in SCA‐strokes (36% [43]) or anterior circulation strokes (13% [34]).

### 4.4 How to Assess the Patient With AIS?

Successful implementation of a more inclusive diagnostic approach has been demonstrated with the STANDING algorithm proposed by Vanni [58, 68] and later validated by Gerlier [69]. The four‐step algorithm starts by identifying any form of nystagmus—including positional—and then assessing the severity of imbalance in those cases without positional nystagmus (indicating BPPV) or spontaneous/gaze‐evoked nystagmus. Patients remaining without a clear peripheral explanation are considered worrisome. Across studies in emergency settings, STANDING achieved sensitivities of 84–100% and specificities of 87%–94% for detecting central causes of acute vertigo/dizziness or imbalance, while also reducing unnecessary neuroimaging and hospitalization rates [58, 68, 69, 70]. These findings underscore that focusing early on imbalance and gait assessment, after resolving for peripheral causes of nystagmus, is an effective and reproducible strategy to identify central pathology in acutely dizzy patients. Likewise, the use of the graded truncal instability (GTI) rating [71] may be considered in those patients without nystagmus instead of HINTS(+). An AVS with severe (grade 3) truncal instability (i.e., inability to stand/sit unassisted) is highly predictive for a central (ischemic) cause (specificity = 99.1% [98.0%–100.0%]) as recently summarized in a meta‐analysis [71], but the sensitivity remains low (44% [34.3%–53.7%]) for a stroke. Considering either grade 2 (inability to walk independently) or grade 3 GTI as central signs, specificity (82.7% [CI = 71.6%–93.8%]) and sensitivity (70.8% [59.3%–82.3%]) are moderate. Thus, while such a graded GTI‐rating does not have a diagnostic accuracy as high as HINTS(+) or STANDING, it is a valuable addition in those dizzy/ataxic patients with no nystagmus. However, in a recent study focusing on patients with acute isolated vertigo/dizziness and mild to moderate symptom burden, clinical gait and stance tests including timed up‐and‐go, functional gait assessment and GTI did not reliably differentiate between peripheral and central origin [72], emphasizing the importance of combining different diagnostic tests in these patients. Complementary to clinical tests for postural control and truncal instability, factors such as timing of symptom onset (acute vs. gradual), associated triggers and accompanying cardiovascular risk (e.g., age, hypertension, dyslipidemia) may be suitable indicators for risk prediction in stroke‐related AIS.

Importantly, presenting ocular motor and vestibular findings may change over time, impacting diagnostic accuracy. For example, in a case series with lateral medullary strokes or ponto‐medullary strokes, initial nystagmus intensity frequently lessened substantially within the frist 24 h [37]. Thus, depending on the timing of the clinical examination, an AVS presentation may shift to an AIS presentation. This emphasizes the value of early (i.e., within the first 24 h) clinical assessment, as proposed by the vascular vertigo diagnostic criteria published [73].

### 4.5 Limitations

This review has several limitations. Classification of patients was constrained by the level of detail reported in the included studies. Findings, particularly the absence of findings or the intended meaning of reported terms, had to be interpreted to the best of our judgment. Studies enrolling patients based on their presenting symptoms typically provided the least detailed clinical information, whereas those restricted to highly specific conditions offered more granular data but at the cost of representativeness. Furthermore, we identified partially overlapping data sets in two pairs of studies ([13, 31] and [51, 52]), but were unable to identify duplicates based on information provided. Albeit in these studies different research questions were addressed, we may not exclude that some patients were counted twice in our analysis. Despite these limitations, this review offers, to our knowledge, the first systematic synthesis focused on the epidemiology and clinical characteristics of the AIS.

## 5 Conclusion

The acute imbalance syndrome (AIS), defined by vertigo/dizziness and/or imbalance in the absence of nystagmus, represents a subtype of AVS as currently defined by ICD‐11, where bedside algorithms such as HINTS(+) are not validated and may be misleading. In our meta‐analysis, almost 50% of all central AVS cases presented as AIS. In AVS with nystagmus, the AICA and PICA territory was proportionally more affected than in AIS, while SCA and PCA territories tended to be relatively more often involved in AIS. Taken together, these findings emphasize the importance of selecting bedside testing appropriately, focusing on other algorithms such as STANDING or GTI in AIS. Importantly, patients presenting as AIS have a distinct (and broader) differential diagnosis than those with AVS with nystagmus. These differences arise mostly from the distribution of noncentral causes, with a much higher fraction of nonvestibular disorders such as dysautonomia or medication side effects and virtually no AUVP. Promoting the awareness of AIS in the ED and its distinct diagnostic approach should be prioritized to further improve the management of this relevant subcohort of patients.

## Author Contributions

**Andreas Zwergal:** writing – review and editing, investigation, validation. **Konstantin Schmidt:** data curation, investigation, writing – review and editing, formal analysis. **Alexander Andrea Tarnutzer:** conceptualization, methodology, data curation, investigation, validation, formal analysis, supervision, writing – original draft, writing – review and editing. **Diego Kaski:** investigation, validation, writing – review and editing.

## Funding

The authors have nothing to report.

## Ethics Statement

Being a systematic review of literature, no ethical approval or consent was necessary.

## Conflicts of Interest

The authors declare no conflicts of interest.

## Data Availability

Source data used for the systematic review will be made available to others upon request to the corresponding author.

## Appendix 1 Electronic Search Strategy, Coding‐Scheme for the Systematic Review and Data Analysis

The search strategy was designed by a neurologist with relevant domain expertise in neuro‐otology (AAT). We searched MEDLINE and Embase for English‐language articles, using the following strategies with the following components: (1) gait imbalance/ataxia, (2) epidemiology, clinical presentation and differential diagnosis, (3) acute imbalance syndrome (ischemic stroke, acute peripheral vestibulopathy) and (4) acute presentation. We also performed a manual search of reference lists from eligible articles and contacted corresponding authors where necessary. We did not seek to identify research abstracts from meeting proceedings or unpublished studies.

AGGREGATED/COMPOSITE VERSION (December 5th, 2024) [PubMed 3287 abstracts; EMBASE 2457 abstracts]

((dizz*[tiab] OR vertigo*[tiab] OR ataxia[tiab] OR imbalance[tiab]) AND (origin[tiab] OR spectrum[tiab] OR epidemiolog*[tiab] OR prevalence[tiab] OR frequency[tiab] OR incidence[tiab] OR distribution*[tiab] OR prodrom*[tiab] OR diagnos*[tiab] OR manifestation*[tiab] OR clinical feature*[tiab] OR symptom*[tiab] OR “physical examination”[mh] OR “medical history taking”[mh] OR physical exam*[tiab] OR “diagnostic tests, routine”[mh] OR professional competence[mh] OR “sensitivity and specificity”[tiab] OR “sensitivity and specificity”[mh] OR “reproducibility of results”[mh] OR “observer variation”[mh] OR “decision support techniques”[mh]) AND (thiamine[tiab] OR multiple sclerosis[tiab] OR intoxicat*[tiab] OR vestibulopathy[tiab] OR labyrin*[tiab] OR vestibular neuritis[tiab] OR vestibular neuronitis[tiab] OR vestibular syndrome[tiab] OR cerebrovascular[tiab] OR stroke*[tiab] OR cerebell*[tiab] OR hemorrhag*[tiab] OR haemorrhag*[tiab] OR vertebrobasilar insufficiency[tiab] OR TIA[tiab] OR transient ischemic attack[tiab]) AND (acute[tiab] OR new‐onset[tiab] OR emerg*[tiab] OR sudden[tiab]) AND (english[la]) AND 2000:2024[dp])

DISAGGREGATED VERSION [to demonstrate overall structure] (December 5th, 2024)

(dizz*[tiab] OR vertigo*[tiab] OR ataxia[tiab] OR imbalance[tiab])

AND

(origin[tiab] OR spectrum[tiab] OR epidemiolog*[tiab] OR prevalence[tiab] OR frequency[tiab] OR incidence[tiab] OR distribution*[tiab] OR prodrom*[tiab] OR diagnos*[tiab] OR manifestation*[tiab] OR clinical feature*[tiab] OR symptom*[tiab] OR “physical examination”[mh] OR “medical history taking”[mh] OR physical exam*[tiab] OR “diagnostic tests, routine”[mh] OR professional competence[mh] OR “sensitivity and specificity”[tiab] OR “sensitivity and specificity”[mh] OR “reproducibility of results”[mh] OR “observer variation”[mh] OR “decision support techniques”[mh])

AND

(thiamine[tiab] OR multiple sclerosis[tiab] OR intoxicat*[tiab] OR vestibulopathy[tiab] OR labyrin*[tiab] OR vestibular neuritis[tiab] OR vestibular neuronitis[tiab] OR vestibular syndrome[tiab] OR cerebrovascular[tiab] OR stroke*[tiab] OR cerebell*[tiab] OR hemorrhag*[tiab] OR haemorrhag*[tiab] OR vertebrobasilar insufficiency[tiab] OR TIA[tiab] OR transient ischemic attack[tiab])

AND

(acute[tiab] OR new‐onset[tiab] OR emerg*[tiab] OR sudden[tiab])

AND humans[mh]

AND english[la]

AND 2000:2024[dp]

**For Embase (search performed Decemer 5th 2024, 2457 citations):**

(dizz*:ab,ti OR vertigo*:ab,ti OR ataxia:ab,ti OR imbalance:ab,ti) AND (origin:ab,ti OR spectrum:ab,ti OR epidemiolog*:ab,ti OR prevalence:ab,ti OR frequency:ab,ti OR incidence:ab,ti OR distribution*:ab,ti OR prodrom*:ab,ti OR diagnos*:ab,ti OR manifestation*:ab,ti OR ‘clinical features’:ab,ti OR ‘clinical feature’:ab,ti OR symptom*:ab,ti OR ‘physical examination’/de OR ‘physical exam’:ab,ti OR 'diagnostic test'/de OR ‘diagnostic tests, routine’/de OR ‘professional competence’/de OR ‘sensitivity and specificity’:ab,ti OR ‘sensitivity and specificity’/de OR ‘reproducibility of results’/de OR ‘observer variation’/de OR ‘decision support techniques’/de) AND (‘thiamine‘:ab,ti OR ‘multiple sclerosis’:ab,ti OR intoxicat*:ab,ti OR ‘vestibulopathy’:ab,ti OR labyrin*:ab,ti OR ‘vestibular neuritis’:ab,ti OR ‘vestibular neuronitis’:ab,ti OR ‘vestibular syndrome’:ab,ti OR cerebrovascular:ab,ti OR stroke*:ab,ti OR cerebell*:ab,ti OR hemorrhag*:ab,ti OR haemorrhag*:ab,ti OR ‘vertebrobasilar insufficiency’:ab,ti OR tia:ab,ti OR ‘transient ischemic attack’:ab,ti OR ‘transient ischaemic attack’:ab,ti) AND (‘acute’:ab,ti OR ‘new‐onset’:ab,ti OR emerg*:ab,ti OR ‘sudden’:ab,ti) AND [1‐1‐2000]/sd AND [English]/lim AND ([article]/lim OR [article in press]/lim OR [data papers]/lim) AND [humans]/lim

### Inclusion and Exclusion Rules for Abstracts & Full‐Text Manuscripts

All gathered literature was subject to title/abstract screening by two independent reviewers (AAT/KS). Abstract review coding rules are provided below. Full‐text screening was applied to all citations considered eligible or possibly eligible by at least one reviewer. Two independent reviewers (AAT/KS) determined whether full‐text manuscripts are eligible and, if not, provided a reason for exclusion (see full‐text review coding rules below). Differences were resolved by discussion and consensus. KS and AAT completed a hand search of the reference lists of selected articles for additional citations. For citations identified by hand search, the full process was repeated iteratively until no additional manuscripts were found for inclusion. Inter‐rater agreement on full‐text inclusion was calculated using Cohen's kappa [21].

### Abstract Review Coding Rules

1. Coding status options are “Yes”, “No”, “Maybe.” We will review full text of “Yes” and “Maybe.”
2. Err on the side of “Maybe” if there is doubt about a “No”; this is more conservative.
3. If there is only a title, exclude it only if you feel confident; otherwise code it as “Maybe.”
4. Each “No” should be coded with a reason for exclusion.
5. Reasons for exclusion are listed below 0–7. Go through them in order from 0 to 7 for each abstract, coding the first reason for exclusion only, not multiple reasons for exclusion. Only code “0” for “not English” if you are sure it is “not English.”
6. Two independent raters (AAT/KS) will code reason for exclusion, but we will *not* mandate agreement on exclusion reason at the abstract level
7. Occasionally an abstract seems inappropriate for another reason. In such cases, code as “other.” There should be few abstracts coded as “other.”

### Abstract Reasons for Exclusion

**Table:**

0	Not English	Manuscript is not in English
1	No data	Review paper; no original patient data
2	Not imbalance	No reasonable prospect that the study includes data about imbalance, ataxia or vertigo/dizziness
3	Not acute	No reasonable prospect that the study includes data about *acute* (< 72 h) dizziness, vertigo or imbalance
4	Not epidemiology	No reasonable prospect that the study includes data about the frequency, spectrum/differential diagnosis or clinical presentation in acute central (specifically stroke) or peripheral (specifically vestibular neuritis) disorders
5	< 5 cases	Fewer than 5 subjects (total participants reported, including cases and controls)
6	Abstract only	Only abstract available (from poster presentation or talk at conference)
7	Other	Any other reason abstract is not included

### Full‐Text Review Coding Rules

1. Coding status options are “Yes” or “No.”
2. Each “No” should be coded with a reason for exclusion.
3. Reasons for exclusion are listed below 0–6. Go through them in order from 0 to 6 for each full manuscript, coding the first reason for exclusion only, not multiple reasons for exclusion.
4. Two independent raters (AAT/KS) will code reason for exclusion, and we will mandate agreement on exclusion reason at the manuscript level.
5. Coding differences will be adjudicated or consensus will be developed through dialog.

### Full‐Text Reasons for Exclusion

**Table:**

0	Not English	Manuscript is not in English
1	No data	Review paper; no original patient data
2	Not imbalance	No reasonable prospect that the study includes data about imbalance, ataxia or vertigo/dizziness
3	Not acute	No reasonable prospect that the study includes data about *acute* (< 72 h) dizziness, vertigo or imbalance
4	Not epidemiology	No reasonable prospect that the study includes data about the frequency, spectrum/differential diagnosis or clinical presentation in acute central (specifically stroke) or peripheral (specifically vestibular neuritis) disorders
5	< 5 cases	Fewer than 5 subjects (total participants reported, including cases and controls)
6	Abstract only	Only abstract available (from poster presentation or talk at conference)

### Search Results

Our search identified 3601 unique citations, of which 3171 (88.1%) were excluded at the abstract level (see PRISMA flow chart). We did not demand concordance on reason for abstract exclusion, but, amongst those abstracts with concordant reasons for exclusion (66.2%, *n* = 2101), the distribution was as follows: 40.4% had fewer than 5 subjects studied, 13.0% had no original data, 8.0% were not about vertigo, dizziness or imbalance, 3.1% did not include data about acute (< 72 h) vertigo, dizziness or imbalance, and 1.8% were not about the epidemiology, frequency/differential diagnosis or clinical presentation.

We sought to examine 430 full manuscripts. After initial screening, there were a total of 17 disagreements (4.0%) about study inclusion for the two reviewers (KS and AAT (search period: 2000–2024), kappa = 0.88). These differences were resolved by discussion and, if needed, adjudication by a third reviewer. Overall agreement on reason for exclusion was 93.1%. We demanded concordance on reason for full‐text exclusion and resolved differences by discussion.

At the end of our full‐text review, 341 studies were excluded and 89 were considered eligible (see PRISMA flow chart). These eligible studies represented 2.5% of the total (*n* = 3601). Amongst all full‐text manuscripts excluded (79.3%), the distribution of reasons for exclusion was as follows: were not about vertigo, dizziness or imbalance (49.8%), were not about the epidemiology, frequency/differential diagnosis or clinical presentation (14.9%), did not include data about acute (< 72 h) vertigo, dizziness or imbalance (10.0%), contained no original data (1.9%), had fewer than 5 subjects studied (1.6%), and were conference abstracts only (1.2%).

## Appendix 2

### Contents

**TABLE A1:** Studies included in the systematic review (*n* = 40).

Author (citation)	Year	Total dizziness sample (studied, % females)	Definition of total sample/Dizzy sample	Definition of AVS/AIS provided/Applied in study	Population (Study Focus)	Setting (country)	Diagnostic testing performed	Data collection (source, analysis)	Mean age (standard deviation, range) [years]	Special comments
Ammar et al. [24]	2017	NR (521, 58%)	ED presentation due to “dizziness, spinning, disequilibrium, lightheadedness, giddiness, or imbalance”	None	Acute dizziness or imbalance with or without nystagmus, central vs. noncentral causes	ED, single community hospital (USA)	MRI or CT in some patients. No information on bedside testing	Retrospective, likely consecutive (ED, based on ICD‐9 codes, cross‐sectional)	49.3 (15.1, NR)	
Braun et al. [25]	2011	NR (12, 42%)	Admitted to ENT ward with diagnosis of peripheral‐origin acute dizziness or imbalance, but with subsequent MRI‐positive lesions	None	Acute dizziness or imbalance with or without nystagmus due to stroke	ENT‐ward, single reference hospital (Austria)	CCT and MRI‐DWI in all. Neurologic examination in all	Retrospective, nonconsecutive (ENT ward, based on ICD‐10 codes, cross‐sectional)	59 (17.7, 25–76)	
Carmona et al. [11]	2023	NR (95, 47%)	ED presentation due to acute vertigo	AVS: “Sudden onset, continuous vertigo lasting > 24 h with associated nystagmus, nausea, and vomiting, all of which are worsened with head movement.”	Acute vertigo and truncal ataxia presenting to the ED: fraction of patients without concurrent nystagmus, central vs. noncentral causes	ED, two reference hospitals (Argentina, UK)	MRI‐DWI in all. Neurologic and neuro‐otologic examination in all. Graded truncal ataxia rating in all	Retrospective, nonconsecutive (ED, cross‐sectional)	Group A (Argentina): 58 (8.3, 30–80) Group B (UK): 57 (16.5, 25–83)	
Chase et al. [26]	2014	NR (473, 60%)	ED presentation due to acute dizziness	None	Acute dizziness with or without nystagmus, stroke vs. nonstroke causes	ED, three reference hospitals (USA)	MRI or CCT in all. Standardized neurologic examination including gait and eye movements	Prospective, nonconsecutive (ED, observational)	56.7 (19.3, NR)	
Chase et al. [27]	2012	NR (131, 58%)	ED presentation due to acute vertigo	None	Acute vertigo with or without nystagmus, stroke vs. nonstroke causes	ED, one reference hospital (USA)	MRI‐DWI in all. Neurologic examination in all	Retrospective, nonconsecutive (ED, cross‐sectional)	57 (18, NR)	
Choi et al. [28]	2014	132 (34, 24%)	ED presentation with confirmed posterior circulation stroke and vestibular syndrome	None	Isolated AVS amongst posterior circulation stroke	ED, one reference hospital (South Korea)	MRI or CCT in all. Neurologic and neuro‐otologic examination in all Graded truncal ataxia rating in all	Prospective, consecutive (ED, observational)	64.4 (12.4, 30–85)	
Choi et al. [29]	2018	1846 (35, 29%)	Early MRI negative cAVS amongst all PCS	No AVS definition provided. Inclusion criteria were “acute vertigo or dizziness, gait unsteadiness and head motion intolerance.” Prolonged symptom duration (days to weeks) is mentioned.	Acute prolonged vertigo/dizziness with confirmed stroke	ED, two reference hospitals (South Korea)	MRI‐DWI in all. Neurologic and neuro‐otologic examination in all Graded truncal ataxia rating in all	Prospective, consecutive (ED, observational)	63 (13, 31–89)	Note that 4/35 patients had recurrent episodes of transient vertigo/dizziness. Note that the rate off alse‐negative PCS with cAVS was 35/850.
Choi et al. [30]	2017	194 (23, NR)	ED presentation with transient acute vertigo/dizziness and gait imbalance, excluding those with BPPV, nonvestibular causes (e.g., orthostatic hypotension, medication/drug intoxication, cardiac arrhythmia) or a history of multiple sclerosis.	Transient AVS: “rapid onset of vertigo/dizziness, gait unsteadiness, and head motion intolerance, and resolution of vestibular symptoms and signs within 24 h”	Acute transient vestibular syndrome, persistent symptoms when presenting to ED. Central vs. noncentral causes	ED, one reference hospital (South Korea)	MRI‐DWI in all. Neurologic and neuro‐otologic examination in all	Prospective, consecutive (ED, observational)	59.1 (15.8, 23–83)	In another 63 patients vestibular symptoms had already stopped when presenting to the ED.
Comolli et al. [31]	2023	1535 (415, NR)	ED presentation due to acute dizziness, vertigo, imbalance, syncope or presyncope	AVS: “clinical syndrome of acute onset, continuous dizziness lasting days to weeks and generally including features suggestive of new, ongoing vestibular system dysfunction (e.g., vomiting, nystagmus, severe postural instability).” AIS: Patients with symptoms that did not meet definitions for AVS/EVS/CVS and therefore a vestibular syndrome could be excluded were classified as an acute imbalance syndrome (AIS). Patients with dizziness as an isolated symptom and no accompanying symptoms or no nystagmus were, therefore, classified as “AIS.”	AVS or AIS presenting to the ED with a diagnosis that allowed a distinction between central vs. noncentral causes.	ED, one reference hospital (Switzerland)	MRI or CCT in some.	Retrospective, consecutive (ED, cross‐sectional)	55.7 (18.6, 16–98) (for entire cohort of 1535 patients)	Note that another 67 patients with AVS and 293 patients with AIS were excluded as no classification (central vs. noncentral) was possible due to missing/unknown diagnosis.
Deluca et al. [32]	2011	NR (92, NR)	Hospitalization due to posterior circulation stroke	None	Patients with posterior circulation stroke and ataxia with or without nystagmus	Neurology ward, 12 community hospitals (Italy)	MRI‐DWI in all. Ataxia examination including ICARS Ocular motor examination (nystagmus)	Prospective, consecutive (neurology ward, observational)	60.2 (14.9, NR)	
Dieterich et al. [33]	2018	158 (23, 70%)	Hospitalization due to midbrain stroke and acute vertigo/dizziness	None	Patients with AVS and/or ocular motor syndromes due to unilateral midbrain stroke with or without nystagmus	Neurology ward, one reference hospital (Germany)	MRI‐DWI in all. Neurologic and neuro‐otologic examination in all	Retrospective, likely consecutive (neurology ward, cross‐sectional)	56.1 (18.0, 26–84)	No definition for AVS provided, despite its use in the study.
Eguchi et al. [34]	2019	668 (8, 25%)	Patients with acute anterior‐circulation cerebral infarction and acute rotatory. Vertigo/dizziness with nausea or vomiting and unsteady gait with illusory self‐motion	None	Patients with anterior circulation stroke and acute vestibular symptoms with or without nystagmus	Neurology ward, one reference hospital (Japan)	MRI‐DWI in all. Neurologic and neuro‐otologic examination in all	Prospective, consecutive (neurology ward, observational)	66.5 (10.8, 52–82)	
Honda et al. [35]	2014	332 (41, 41%)	ED presentation with acute imbalance, including vertigo and dizziness	None	Patients with isolated acute imbalance and vertigo/dizziness and confirmed stroke with or without nystagmus	ED, one reference hospital (Japan)	MRI‐DWI in all. Neurologic and neuro‐otologic examination in all	Prospective, consecutive (ED, observational)	70.2 (11.2, NR)	
Jo et al. [36]	2019	1925 (468, 56%)	Isolated vertigo/dizziness presenting to ED	None	Patients with isolated acute vertigo/dizziness with or without nystagmus, central vs. noncentral causes	ED, one reference hospital (South Korea)	MRI‐DWI in all. Neurologic examination in all	Retrospective, consecutive (ED, cross‐sectional)	60.8 (14.0, NR)	Only strokes and space‐occupying lesions were included in the central group.
Kattah [37]	2023	161 (13, NR)	ED presentation with acute prolonged vertigo/dizziness and confirmed medullary or pontine stroke	None	Patients with AVS ataxia grade 2/3, medullary or pontine stroke and confirmed ocular lateral deviation (with or without nystagmus)	ED, one reference hospital (USA)	MRI‐DWI in all. Neurologic and neuro‐otologic examination in all.	Prospective, consecutive (ED, observational)	54.8 (15.8, 28–88)	No AVS definition is provided, but likely presence of nystagmus was nonmandatory.
Kerber et al. [38]	2012	595 (13, 31%)	ED presentation with nontraumatic acute intracranial hemorrhage (ICH) and dizziness	None	Patients with acute dizziness due to ICH and NIHSS < 2, with or without nystagmus	ED, one reference hospital (USA)	CCT in all. Neurological examination in all	Retrospective, consecutive (ED, cross‐sectional)	73.2 (10.6, 58–92)	
Kerber et al. [39]	2015	320 (272, 51%)	ED presentation due to acute dizziness	None	Patients with acute dizziness, with or without nystagmus. Stroke vs. nonstroke causes.	ED, one reference hospital (USA)	MRI‐DWI in all. Neurologic and neuro‐otologic examination in all.	Prospective, consecutive (ED, observational)	Group 1 (confirmed stroke) (median, IQR): 60.6 (51.0, 71.3) Group 2 (no stroke) (median, IQR): 56.1 (48.6, 66.5)	
Kim and Kim [40]	2019	NR (23, 57%)	Middle cerebellar peduncle stroke (ischemic/hemorrhagic) on MRI	None	Patients with acute vertigo/imbalance and acute strokes restricted to the middle cerebellar peduncle, with or without nystagmus	ED, one reference hospital (South Korea)	MRI‐DWI in all. Neurologic and neuro‐otologic examination in all.	Prospective, nonconsecutive (ED, observational)	62.0 (10.2, 23–85)	
Kmetonyova et al. [41]	2023	695 (119, 62%)	ED presentation due to acute dizziness and gait instability	None	Patients with acute dizziness and gait instability, with or without nystagmus. Stroke vs. nonstroke causes.	ED, one reference hospital (Czech Republic)	MRI‐DWI in all. Neurologic and neuro‐otologic examination in all.	Prospective, likely consecutive (ED, observational)	Group 1 (no ischemic lesion): 52.1 (14.9, NR) Group 2 (confirmed stroke): 66.1 (15.4, NR)	21 cases with no definitive diagnosis and normal brain MRI were excluded
Lee and Baloh [42]	2005	364 (28, 39%)	ED presentation due to sudden deafness, vertigo and confirmed stroke	None	Acute audiovestibular loss and confirmed vertebrobasilar stroke with or without nystagmus	ED, one reference hospital (USA)	MRI‐DWI in all. Neurologic and neuro‐otologic examination in all.	Prospective, consecutive (ED, observational)	61.7 (13.8, 28–93)	
Lee and Kim [43]	2013	41 (31, NR)	Patients with isolated SCA infarction presenting to ED	None	Patients with acute vertigo or dizziness and confirmed SCA stroke, with or without nystagmus	ED, one reference hospital (South Korea)	MRI‐DWI in all. Neurologic and neuro‐otologic examination in all.	Prospective, nonconsecutive (ED, observational)	Group 1 (patients with nystagmus): 62.5 (13.8, NR) Group 2 (patients without nystagmus): 65.1 (11.8, NR)	
Lee et al. [44]	2023	8 (5, 60%)	Patients with (hemi)nodular stroke and acute vertigo	AVS: “acute onset of vertigo or dizziness with nausea or vomiting, head‐motion intolerance, and unsteadiness”	Patients with acute vertigo and confirmed (hemi)nodular stroke, with or without nystagmus	ED, one reference hospital (South Korea)	MRI‐DWI in all. Neurologic and neuro‐otologic examination in all.	Retrospective, consecutive (ED, cross‐sectional)	60.2 (9.4, 49–73)	All 5 patients had persistent symptoms for > 24 h.
Li et al. [45]	2024	NR (109, 59%)	ED presentation due to isolated vertigo or dizziness.	None	Patients with isolated acute vertigo or dizziness with or without nystagmus, central vs. noncentral causes.	ED, one reference hospital (China)	MRI‐DWI in all. Neurologic and neuro‐otologic examination in all.	Retrospective, nonconsecutive (ED, cross‐sectional)	62.4 (13.0, NR)	Note that patients with dizziness or vertigo that may also be caused by other diseases (such as anemia, hyperthyroidism, fever, or hypotension) were excluded
Liu et al. [46]	2024	NR (121, 34%)	ED presentation due to acute vertigo, dizziness or imbalance	AVS: “new spontaneous and persisting vertigo, dizziness or imbalance (lasting for > 24 h)”	Patients with AVS with or without nystagmus, central vs. noncentral causes	ED, one reference hospital (China)	MRI‐DWI in all. Neurologic and neuro‐otologic examination in all.	Prospective, consecutive (ED, observational)	55.0 (40.0–64.0) (median, IQR)	
Machner et al. [19]	2020	610 (242, NR)	Patients presenting to the ED with dizziness/vertigo and admitted to one of the hospital's neurological wards	AVS: “Vestibular symptoms and spontaneous nystagmus, continuously present at ED presentation.” AIS: “Acute onset of an unsteadiness in stance and gait, still persistent at ED presentation, no spontaneous nystagmus.”	Patients with AVS or AIS, central vs. noncentral causes	ED, one reference hospital (Germany)	MRI or CCT in all. Neurologic and neuro‐otologic examination in all.	Retrospective, consecutive (ED, observational)	NR	AVS with sx‐onset > 72 h, w/o MRI, and sx‐duration < 24 h were excluded. Also those with dizziness due to a clear medical cause or being discharged from ED were excluded
Mandge et al. [47]	2020	505 (14, 43%)	ED presentation due to acute vertigo or dizziness	None	Patients with acute vertigo or dizziness due to central causes, with or without nystagmus	ED, one municipal hospital (USA)	MRI‐DWI in all. Neurologic and neuro‐otologic examination in all.	Retrospective, consecutive (ED, cross‐sectional)	59.4 (15.4, NR)	Nystagmus was reported only for the 14 central‐vestibular cases, thus all 162 peripheral‐vestibular cases were excluded
Moon et al. [48]	2009	NR (8, 38%)	ED presentation due to acute vertigo and imbalance, receiving a diagnosis of isolated nodular infarction	None	Patients with acute vertigo or imbalance due to central causes (isolated nodular infarction) with or without nystagmus	ED, six reference hospitals (South Korea)	MRI‐DWI in all. Neurologic and neuro‐otologic examination in all.	Retrospective, nonconsecutive (ED, cross‐sectional)	60.1 (12.4, 37–75)	Symptom duration at ED presentation not reported, thus unclear if > 24 h duration.
Morita et al. [49]	2011	NR (8, 63%)	ED presentation due to severe isolated vertigo or dizziness and initially false‐negative MRI‐DWI in acute cerebellar stroke	None	Patients with isolated central vertigo or dizziness for > 24 h duration with or without nystagmus	ED, one reference hospital (Japan)	MRI‐DWI in all. Neurologic and neuro‐otologic examination in all.	Retrospective, nonconsecutive (ED, cross‐sectional)	71.5 (7.3, 61–84)	
Navi et al. [50]	2012	NR (907, 58%)	ED presentation due to acute dizziness, vertigo, imbalance or disequilibrium	None	Patients with acute vertigo/dizziness/imbalance, with or without nystagmus. Central vs. noncentral causes	ED, one reference hospital (USA)	MRI or CCT in some (321/907). Neurological examination in some (neurology consultation obtained for 180 patients)	Retrospective, consecutive (ED, cross‐sectional)	59 (19, NR)	Distinction for nystagmus reporting was made between central «serious neurologic disorders» and «other diagnoses» only, with the latter category including mostly noncentral causes, but also migraine and concussions were assigned to this category
Nham et al. [51]	2023	NR (205, 43%)	ED presentation due to acute vertigo and/or imbalance	None	Patients with AVS with or without nystagmus. Stroke vs. acute unilateral vestibulopathy	ED, two reference hospitals (Turkey, Australia)	MRI or CCT in some (brain imaging in all strokes, but only in part of AUVP cases). Neurologic and neuro‐otologic examination in all.	Prospective, consecutive (ED, observational)	Group 1 (strokes): 67.0 (12.3, NR) Group 2 (AUVP): 56.9 (17.0, NR)	No definition of AVS provided, but both cases with and without nystagmus were considered to have AVS
Nham et al. [52]	2023	1154 (262, 36%)	ED presentation due to acute vertigo or imbalance	AVS definition as proposed by Kerber [53] is used: “AVS is characterized by new‐onset persistent dizziness which distinguishes it from the episodic and chronic syndromes. At the time of an AVS presentation, patients typically have severe and often disabling dizziness, nausea, vomiting, and head motion intolerance. Other criteria for AVS are examination findings of gait unsteadiness and/or nystagmus.”	Patients with AVS with or without nystagmus. Stroke vs. acute unilateral vestibulopathy	ED, one reference hospital (Australia)	MRI or CCT in some (brain imaging in all strokes, but only in part of AUVP cases). Neurologic and neuro‐otologic examination in all.	Prospective, consecutive (ED, observational)	Group 1 (strokes): 67.6 (13.4, NR) Group 2 (AUVP): 55.9 (17.0, NR)	There is potential overlap with another study from Nham et al. [30] as recruiting periods and places were overlapping for these two studies.
Nikles et al. [13]	2024	1647 (115, NR)	ED presentation due to acute vestibular symptoms (dizziness, vertigo, and imbalance)	AVS defined as “consisting of continuous dizziness/vertigo, nausea, vomiting, nystagmus, gait disturbance, and motion intolerance.”	Patients with acute vestibular symptoms and MRI‐confirmed stroke (PCS vs. ACS), presenting on ED, with or without nystagmus	ED, one reference hospital (Switzerland)	MRI‐DWI in all. Neurologic and neuro‐otologic examination in all.	Prospective, consecutive (ED, observational)	67.3 (15.6, 24–93)	Note that single patients could have strokes in more than one vascular territory, thus the total number of strokes (*n* = 143) is larger than the number of patients (*n* = 115). Note also that 7 patients with combined ACS and PCS were excluded.
Ogawa et al. [54]	2016	NR (11, 27%)	Acute presentation due to vertigo or dizziness, initially misdiagnosed as of peripheral origin	None	Patients with acute vertigo/dizziness and MRI‐confirmed cerebellar stroke, with or without nystagmus	ENT‐ward, one reference hospital (Japan)	MRI or CCT in all. Neurologic and neuro‐otologic examination in all.	Retrospective, nonconsecutive (ENT ward, cross‐sectional)	66.4 (13.1, 35–83)	
Ohle et al. [55]	2024	2618 (2078, 41%)	ED presentation due to acute vertigo, dizziness or imbalance	None	Patients with acute vertigo/dizziness/imbalance, with or without nystagmus. Serious vs. nonserious causes	ED, three reference hospitals (Canada)	MRI or CCT in some (699/2078).	Prospective, consecutive (ED, observational)	NR (NR, NR)	Serious causes included a diagnosis of stroke, TIA, vertebral artery dissection, or brain tumor.
Sandlund et al. [56]	2019	NR (3613, 57%)	ED presentation due to acute vertigo or dizziness	“AVS was defined as new (since ≤ 72 h) ongoing and continuous dizziness fulfilling at least two of the following criteria: (a) nystagmus (b) nausea or vomiting (c) gait or balance disturbance during physical examination, and (d) discomfort with head movement.”	Patients with AVS with or without nystagmus.	ED, one reference hospital (Sweden)	MRI or CCT in some (699/3613).	Prospective, consecutive (ED, observational)	Period 1 (2012–2014): 63.8 (18.9, NR) Period 2 (2016–2017): 62.6 (19.4, NR)	No specific diagnoses were provided, thus no distinction between central vs. noncentral AVS/AIS could be made. Effect of an acute dizziness management algorithm (period 1 vs. 2) was studied.
Saro‐Buendia et al. [57]	2023	NR (85, 35%)	ED presentation due to acute dizziness and diagnosis of PCS	AVS defined as “abrupt monophasic dizziness associated with nausea or vomiting, gait instability, spontaneous nystagmus, and intolerance to cephalic movements”	Patients with AVS or AIS due to PCS. Presence of nystagmus was assessed	ED, one reference hospital (Spain)	MRI‐DWI in all. Neurologic and neuro‐otologic examination in all.	Retrospective, nonconsecutive (ED, cross‐sectional)	Median and IQR: 73 (64–79)	
Shi et al. [20]	2022	NR (79, NR)	ED presentation due to acute vertigo, dizziness or imbalance and a symptom duration of < 4.5 h and discharge diagnosis of ischemic stroke.	AVS: “Defined as persistent vertigo or dizziness with nystagmus.” AIS: “Defined as persistent dizziness or imbalance without nystagmus.”	Patients with AVS or AIS and confirmed stroke	ED, one reference hospital (China)	MRI or CCT in all. Neurologic and neuro‐otologic examination in all.	Retrospective, consecutive (ED, cross‐sectional)	IVT group: 64.0 (15.1, NR) Non‐IVT group: 67.1 (14.9, NR)	Comparison between those that received intravenous thrombolysis and those that did not was provided as well. Note that 10 patients with acute transient vestibular syndrome were excluded.
Vanni et al. [58]	2017	391 (352, 60%)	ED presentation due to acute vertigo or unsteadiness	None	Patients with acute vertigo or imbalance, with or without nystagmus. Central vs. noncentral causes	ED, one reference hospital (Italy)	MRI or CCT in some (164/352). STANDING algorithm performed in all patients	Prospective, consecutive (ED, observational)	Median and IQR: 59 (43–72)	
Yang et al. [59]	2019	NR (14, 7%)	ED presentations due to acute isolated vertigo	None	Patients with acute vertigo without focal neurologic signs and confirmed PICA stroke, with or without nystagmus.	ED, one reference hospital (South Korea)	MRI‐DWI in all. Neurologic and neuro‐otologic examination in all.	Retrospective, nonconsecutive (ED, cross‐sectional)	63.4 (9.4, NR)	Nystagmus was only reported for the PICA stroke subgroup (14/149 patients with acute vertigo)
Zuo et al. [60]	2018	353 (273, 52%)	ED presentations due to acute isolated vertigo or dizziness	None	Patients with acute vertigo or dizziness without focal neurologic signs, with or without nystagmus. Stroke vs. nonstroke causes	ED, one reference hospital (China)	MRI or CCT in all. Neurologic examination in all	Retrospective, consecutive (ED, cross‐sectional)	67.1 (12.0, NR)	Note that nonstroke cases could be central of origin as well

Abbreviations: ACS, anterior circulation stroke; AIS, acute imbalance syndrome; AUVP, acute unilateral vestibulopathy; AVS, acute vestibular syndrome; CCT, cranial computed tomography; DWI, diffusion‐weighted imaging; ED, emergency department; ICH, intracranial hemorrhage; IQR, interquartile range; IVT, intravenous thrombolysis; MRI, magnetic resonance imaging; NIHSS, National Institutes of Health Stroke Scale; NR, not reported; PCS, posterior circulation stroke; PICA, posterior inferior cerebellar artery.
